# Multiple Myeloma of the Orbit

**DOI:** 10.1155/2012/252310

**Published:** 2012-11-06

**Authors:** Mona Hassan, Zaid Alirhayim, Laila Sroujieh, Syed Hassan

**Affiliations:** Department of Internal Medicine, Henry Ford Hospital, 2799 West Grand Boulevard, Detroit, MI 48202, USA

## Abstract

The authors report a case of a 62-year-old female with history of multiple myeloma who presents with complains of swelling and pain in her right eye. On examination, it was found that she has proptosis, chemosis, and diplopia along with decreased vision. Initial workup and treatment did not yield significant results, eventually she was found to have myelomatous changes in her right orbit on MRI and was diagnosed with multiple myeloma of the orbit which resolved solely with radiation. This case tends to highlight the importance of considering myeloma of the orbit as a very important and early differential diagnosis in a patient with a history of multiple myeloma presenting with a swollen and painful eye.

## 1. Case

**Figure 1:**
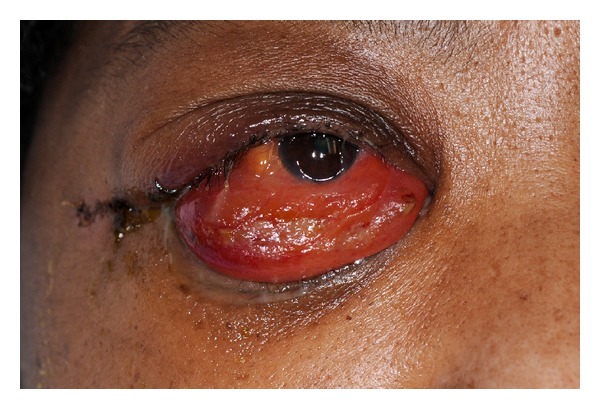
Initial presentation of the right ocular multiple myeloma.

**Figure 2:**
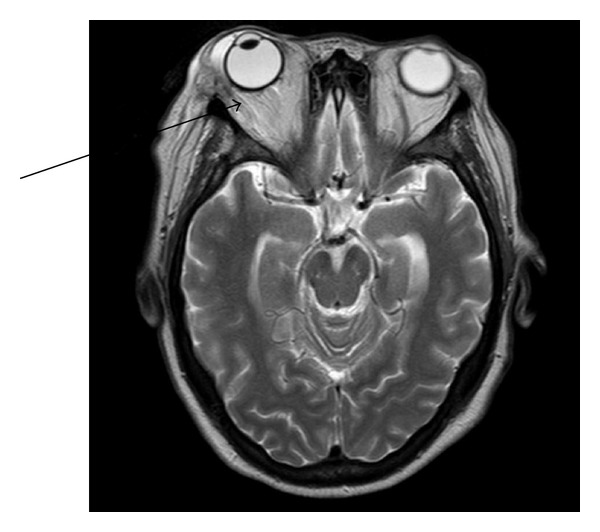
MRI of the orbit showing involvement of the greater wing of the sphenoid along with right orbital extension, involving the lateral rectus muscle and the lateral wall of the orbit as well as a right-sided preseptal soft tissue swelling and thickening.

**Figure 3:**
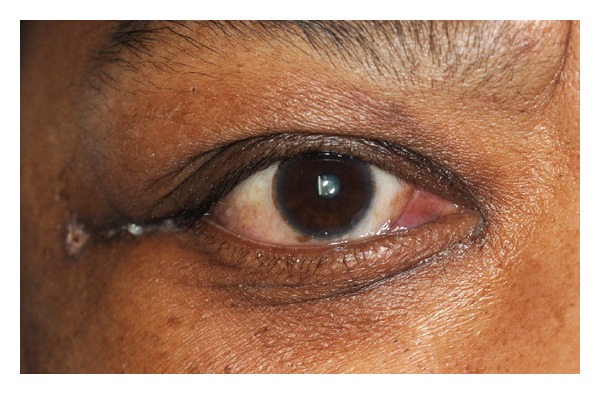
Presentation of the right eye after radiation therapy.

A 62-year-old female patient presented to the emergency department with complains of excruciating lower back pain since two months, and X-ray of the spine was suggestive of osteolytic lesion of the lumbar spine at L-3; this was consistent with her history of multiple myeloma. However, the patient was in remission at the time of presentation. Medication included bortezomib, dexamethasone, and zoledronic acid. She was admitted as inpatient and palliative radiation to the spine was given. On the 5th day of her admission, she started complaining of trouble with vision and pain in the right eye upon horizontal gaze. Closing of the eye alleviates the pain. Diffuse edema of both eyelids was prominent and was tender to palpation. There was no associated erythema of the eyelids or any signs suggestive of preseptal cellulites (Figure 1). Upon further examination it showed proptosis and chemosis of the orbit with some transient episode of diplopia. The visual acuity was 10/20 in the right eye and 20/25 in the left eye. Upon anterior segment examination, there was conjunctival chemosis that is more prominent in the inferotemporal area. This area was not injected and there was no evidence of hemorrhage. The reminder of anterior segment and dilated ophthalmoscopy examination was unremarkable. CT of the orbit showed fluid collection in the right lateral periocular space, which was concerning for a subperiosteal abscess, for which she underwent incision and drainage and was started on metronidazole, cefepime, and vancomycin intravenously along with erythromycin eye drops. Differential at that point also included cavernous sinus thrombosis, but was considered unlikely by our experts in neurology. The patient was not responding to antibiotics and her clinical course was worsening. Finding no improvement in her condition, MRI of the eye was done that was suggestive of myelomatous changes involving the bones of the right orbital socket (Figure 2). With a history of multiple myeloma, myeloma of the orbit was now very high on our differential. Radiation oncology was consulted and she underwent two sessions of stereotactic radiation. With radiation therapy within three days her chemosis and proptosis resolved and the pain in her eye subsided with no visual disturbances (Figure 3). She was then followed outpatient periodically with no recurrence of symptoms. However, she died 4 months later due to complications from her progressive cancer. 

## 2. Discussion

Multiple myeloma of the orbit is a rare, but a serious condition. Involvement of almost every ocular structure has been reported [1–7]. Most common clinical presentation includes proptosis, redness, pain, diplopia, and decreased vision. Proptosis was also noted to be a sign of recurrence of multiple myeloma in patients who are though to be in remission [8]. Unilateral involvement of one eye is more common [9]. Both of these presentations were seen in our case. It is argued that myelomatous changes of the orbit are common, even though clinical presentation is extremely rare [10]. Solitary extramedullary orbital plasmacytoma as an initial presenting feature in a multiple myeloma patient has also been reported [11]. In most of the cases reported, CT scan was the imaging modality of choice, though in our case it was not much helpful and in fact led us away from the diagnosis [12]. Treatment options include systemic chemotherapy and local radiation and often good response to these options is noted [13]. Our case is unique because of its dramatic presentation as seen in the images and quick resolution of the symptoms after a very short duration of radiation therapy.
